# Xylo- and cello-oligosaccharide oxidation by gluco-oligosaccharide oxidase from *Sarocladium strictum* and variants with reduced substrate inhibition

**DOI:** 10.1186/1754-6834-6-148

**Published:** 2013-10-12

**Authors:** Thu V Vuong, Arja-Helena Vesterinen, Maryam Foumani, Minna Juvonen, Jukka Seppälä, Maija Tenkanen, Emma R Master

**Affiliations:** 1 Department of Chemical Engineering and Applied Chemistry, University of Toronto, 200 College Street, Toronto, Ontario, M5S 3E5, Canada; 2 Department of Biotechnology and Chemical Technology, Aalto University, Kemistintie 1 D1, Espoo 02150, Finland; 3 Department of Food and Environmental Sciences, University of Helsinki, P.O. Box 27, Helsinki 00014, Finland

**Keywords:** Gluco-oligosaccharide oxidase, *Sarocladium strictum*, Cello-oligosaccharide, Xylo-oligosaccharide, Substrate specificity, Oxidation, Substrate inhibition, Protein engineering

## Abstract

**Background:**

The oxidation of carbohydrates from lignocellulose can facilitate the synthesis of new biopolymers and biochemicals, and also reduce sugar metabolism by lignocellulolytic microorganisms, reserving aldonates for fermentation to biofuels. Although oxidoreductases that oxidize cellulosic hydrolysates have been well characterized, none have been reported to oxidize substituted or branched xylo-oligosaccharides. Moreover, this is the first report that identifies amino acid substitutions leading to GOOX variants with reduced substrate inhibition.

**Results:**

The recombinant wild type gluco-oligosaccharide oxidase (GOOX) from the fungus *Sarocladium strictum*, along with variants that were generated by site-directed mutagenesis, retained the FAD cofactor, and showed high activity on cello-oligosaccharide and xylo-oligosaccharides, including substituted and branched xylo-oligosaccharides. Mass spectrometric analyses confirmed that GOOX introduces one oxygen atom to oxidized products, and ^1^H NMR and tandem mass spectrometry analysis confirmed that oxidation was restricted to the anomeric carbon. The A38V mutation, which is close to a predicted divalent ion-binding site in the FAD-binding domain of GOOX but 30 Å away from the active site, significantly increased the *k*_cat_ and catalytic efficiency of the enzyme on all oligosaccharides. Eight amino acid substitutions were separately introduced to the substrate-binding domain of GOOX-VN (at positions Y72, E247, W351, Q353 and Q384). In all cases, the *K*_m_ of the enzyme variant was higher than that of GOOX, supporting the role of corresponding residues in substrate binding. Most notably, W351A increased *K*_m_ values by up to two orders of magnitude while also increasing *k*_cat_ up to 3-fold on cello- and xylo-oligosaccharides and showing no substrate inhibition.

**Conclusions:**

This study provides further evidence that *S*. *strictum* GOOX has broader substrate specificity than the enzyme name implies, and that substrate inhibition can be reduced by removing aromatic side chains in the -2 binding subsite. Of the enzyme variants, W351A might be particularly advantageous when oxidizing oligosaccharides present at high substrate concentrations often experienced in industrial processes.

## Background

Recently, a new classification of carbohydrate active enzymes termed auxiliary activities (or AA), was introduced to the carbohydrate-active enzyme database (CAZy; http://www.cazy.org) [1]. Many of the enzymes classified into AA families are carbohydrate oxidases. Well-known examples include cellobiose dehydrogenase (CDH, EC 1.1.99.18, AA3_1) [2], glucose 1-oxidase (EC 1.1.3.4, AA3_2) [3], pyranose 2-oxidase (EC 1.1.3.10, AA3_4) [4], and galactose 6-oxidase (EC 1.1.3.9, AA5_2) [5]. Comparatively few publications describe the activity of gluco-oligosaccharide oxidases (GOOX, EC 1.1.3.-), which are classified as family AA7 enzymes, and exhibit high catalytic activity on oligomeric substrates [6,7].

Early reports of GOOX-T1 from the fungus *Sarocladium strictum* T1 (previously known as *Acremonium strictum* T1 [8]) confirmed oxidation of the hydroxyl group attached to the anomeric carbon of maltose [6]; other analyses revealed even higher activities on cello-oligosaccharides, particularly cellotriose [9,10]. Like other flavin carbohydrate oxidases that target the hydroxyl group of the anomeric carbon, GOOX-T1 is thought to mediate oxidoreductase activity through two half-reactions: 1) oxidation of the reducing sugar to the corresponding lactone, and 2) reduction of molecular oxygen to hydrogen peroxide [11]. Subsequent hydrolysis of the lactone product to the corresponding carboxylic acid may then occur. While the biological function of GOOX is uncertain, hydrogen peroxide generated through carbohydrate oxidation could be used by lignin peroxidases and manganese peroxidase in lignin degradation. From an applied perspective, gluco-oligosaccharide oxidases could provide an alternative to CDHs used in amperometric enzyme biosensors for real-time measurement of cellulase activity on insoluble cellulose [12]. More recent applications of CDH also demonstrate the benefit of carbohydrate oxidation to reduce sugar consumption by lignocellulolytic fungi, thereby maximizing ethanol yields from fermenting microorganisms [13].

The crystal structure of GOOX-T1 reveals a monomeric glycoprotein with a flavin adenine dinucleotide (FAD)-binding domain coordinated by a bi-covalent linkage to H70 (8α-N1-histidyl) and C130 (6-S-cysteinyl); GOOX-T1 is also characterized by having a comparatively open substrate-binding site [14]. Site-directed mutagenesis confirmed the requirement of bi-covalent coordination of FAD for enzyme activity; this unique coordination is also correlated to the relatively high redox potential of GOOX-T1 [14,15]. In our recent study, GOOX-VN from *S*. *strictum* strain CBS 346.70 was recombinantly expressed and biochemically characterized using a range of sugars and oligosaccharides, including cello-oligosaccharides and xylo-oligosaccharides with up to 3 sugar units [7]. Fifteen amino acid differences distinguish GOOX-VN and GOOX-T1: 13 are intrinsic differences in the wild-type gene sequences while 2 (A38V and S388N) arose from random mutations during the construction of the GOOX-VN expression system [7] (Additional file 1: Figure S1). GOOX-VN was found to oxidize xylose as well as xylobiose and xylotriose [7]. Given the high sequence identity between GOOX-VN and GOOX-T1 (97%), and since none of the amino acid substitutions between GOOX-VN and GOOX-T1 are predicted to directly participate in substrate binding, it is likely that GOOX-T1 also oxidizes xylo-oligosaccharides even though xylo-oligosaccharide oxidation by GOOX-T1 has not been reported [7,10]. Notably, resulting enzymatically oxidized oligosaccharides could be used as carbohydrate standards that replaces the comparatively arduous chemical synthesis approach [16], facilitating the characterization of carbohydrate-oxidizing enzymes whose activity can not be easily measured by colorimetric assays.

**Figure 1:**
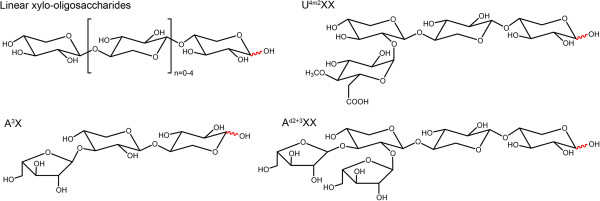
**The structures of xylo**-**oligosaccharides used in this study.** A^3^X, α-L-arabinofuranosyl-(1→3)-β-D-xylopyranosyl-(1→4)-D-xylose; A^d2+3^XX, α-L-arabinofuranosyl-(1→2)-[α-L-arabinofuranosyl-(1→3)]-β-D-xylopyranosyl-(1→4)-β-D-xylopyranosyl-(1→4)-D-xylose; U^4m2^XX, 4-*O*-methyl-α-D-glucopyranosyl uronic acid-(1→2)-β-D-xylopyranosyl-(1→4)-β-D-xylopyranosyl-(1→4)-D-xylose.

To investigate the role of selected amino acids on substrate preference, three amino acids in the GOOX-VN substrate binding site were previously substituted to corresponding residues in chito-oligosaccharide oxidase (ChitO) from *Fusarium graminearum*[15] or carbohydrate oxidase from *Microdochium nivale*[17], which show 45% and 42% sequence identity to GOOX-VN, respectively [7]. Of these, Y300A nearly doubled *k*_cat_ values for oligosaccharides while also increasing corresponding *K*_m_ values [7]. The current study describes a more comprehensive assessment of substrate preference and catalysis by GOOX-VN by 1) constructing eight additional amino acid substitutions within the substrate binding site of this enzyme, 2) generating V38A and N388S substitutions that convert GOOX-VN to the wild-type GOOX sequence, and 3) using several oligosaccharides, including branched xylo-oligosaccharides (Figure 1) to characterize the catalytic efficiency, substrate selectivity and substrate inhibition of GOOX-VN enzyme variants. These analyses confirmed comparable kinetic efficiencies on cello-oligosaccharides and xylo-oligosaccharides, suggesting that gluco-oligosaccharide oxidases characterized to date have broader substrate specificity than the enzyme name implies. This study also identified enzyme variants with high catalytic activity but lower substrate inhibition, which could improve oligosaccharide oxidation at high substrate concentrations often experienced in industrial bioprocesses.

## Results and discussion

### Protein expression and biophysical characterization

**Figure 2:**
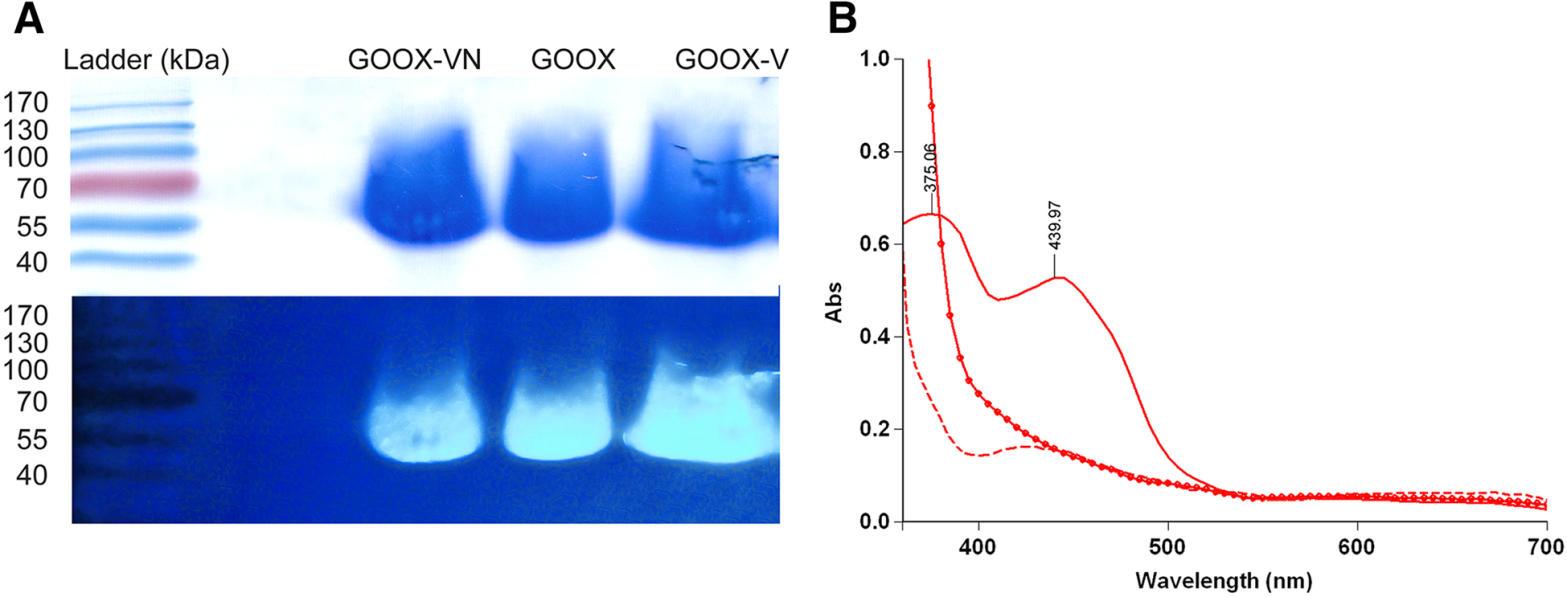
**The presence of the covalent FAD cofactor in GOOX. (A)**: An SDS-PAGE of GOOX and mutant variants stained by Coomassie blue (upper) and under 254 nm transillumination (lower), which shows intrinsic fluorescence of the covalently-bound FAD upon acidification; protein samples were overloaded to facilitate the detection of FAD intrinsic fluorescence. **(B)**: UV–VIS scanning of GOOX, showing two automatically-determined maxima of 375 and 440 nm, which are similar to the absorbance (Abs) peaks of GOOX-T1 at 380 and 444 nm, respectively [10]; the 440-nm peak disappeared when the enzyme was reduced by 50 mM sodium hydrosulfite (dotted solid line) or by 200 mM cellobiose (dash line).

Recombinantly expressed GOOX-VN and enzyme variants were purified to more than 95% homogeneity by affinity chromatography (Figure 2A, Additional file 2: Figure S2). Amino acid substitutions did not affect protein yields, and in general, between 5 and 10 mg/L of purified protein were recovered. The observed mass of all enzymes was approximately 70 kDa (Additional file 2: Figure S2), suggesting that glycosylation could account for approximately 20% of the protein, which is similar to the mass percentage of carbohydrates in glucose oxidase [18]. Notably, the deglycosylation of GOOX-VN by PNGaseF, generated a band at about 56 kDa on SDS-PAGE gels [7], but this deglycosylation did not affect the activity or substrate specificity of the enzyme (Table 1).

**Table 1:** **Effect of glycosylation on GOOX activity on cello**- **and xylo**-**oligosaccharides**

	**Specific activity of GOOX (μmol min**^**-1 **^**mg**^**-1**^)*	
**Substrate**	**Deglycosylated**	**Glycosylated**
Cellobiose	9.03 ± 0.05	9.20 ± 0.11
Xylobiose	12.55 ± 0.11	12.72 ± 0.13
Cellopentaose	8.19 ± 0.03	8.29 ± 0.13
Xylopentaose	11.91 ± 0.09	11.93 ± 0.01

*Specific activity (μmol min^-1^ mg^-1^) of deglycosylated and glycosylated GOOX was measured at 37°C for 15 min using 0.5 mM of each substrate.

None of the amino acid substitutions appeared to affect FAD binding, as assessed by fluorescence detection (Figure 2A) and UV–VIS scanning (Figure 2B). Enhancement of fluorescence following performic acid oxidation is a convenient method for detecting the presence of 8α-S-cysteinyl riboflavins [19]. Since pre-treatment of SDS-PAGE gels with performic acid did not increase the fluorescence measured from GOOX-VN or any of the enzyme variants, one of the covalent linkages to the FAD cofactor is likely 6-S-cysteinyl as seen in GOOX-T1 structures [14,20]. Moreover, because the flavinylation process is thought to promote proper protein folding [14], detection of the FAD cofactor suggests that enzyme variants have assumed the correct protein conformation.

### Confirming the regioselectivity of gluco-oligosaccharide oxidases

To date, very few studies have confirmed the position of hydroxyl groups oxidized by family AA7 gluco-oligosaccharide oxidases. Lee at al. [6] used ^13^C and ^1^H NMR to confirm that GOOX-T1 targets the hydroxyl group of the anomeric carbon, however, only maltose was used in their analysis. Since gluco-oligosaccharide oxidase activity is higher on cello-oligosaccharides and xylo-oligosaccharides than maltose [6,7], ^1^H NMR was used here to evaluate the effect of sugar type and linkage on the regio-selectivity of GOOX enzymes.

**Figure 3:**
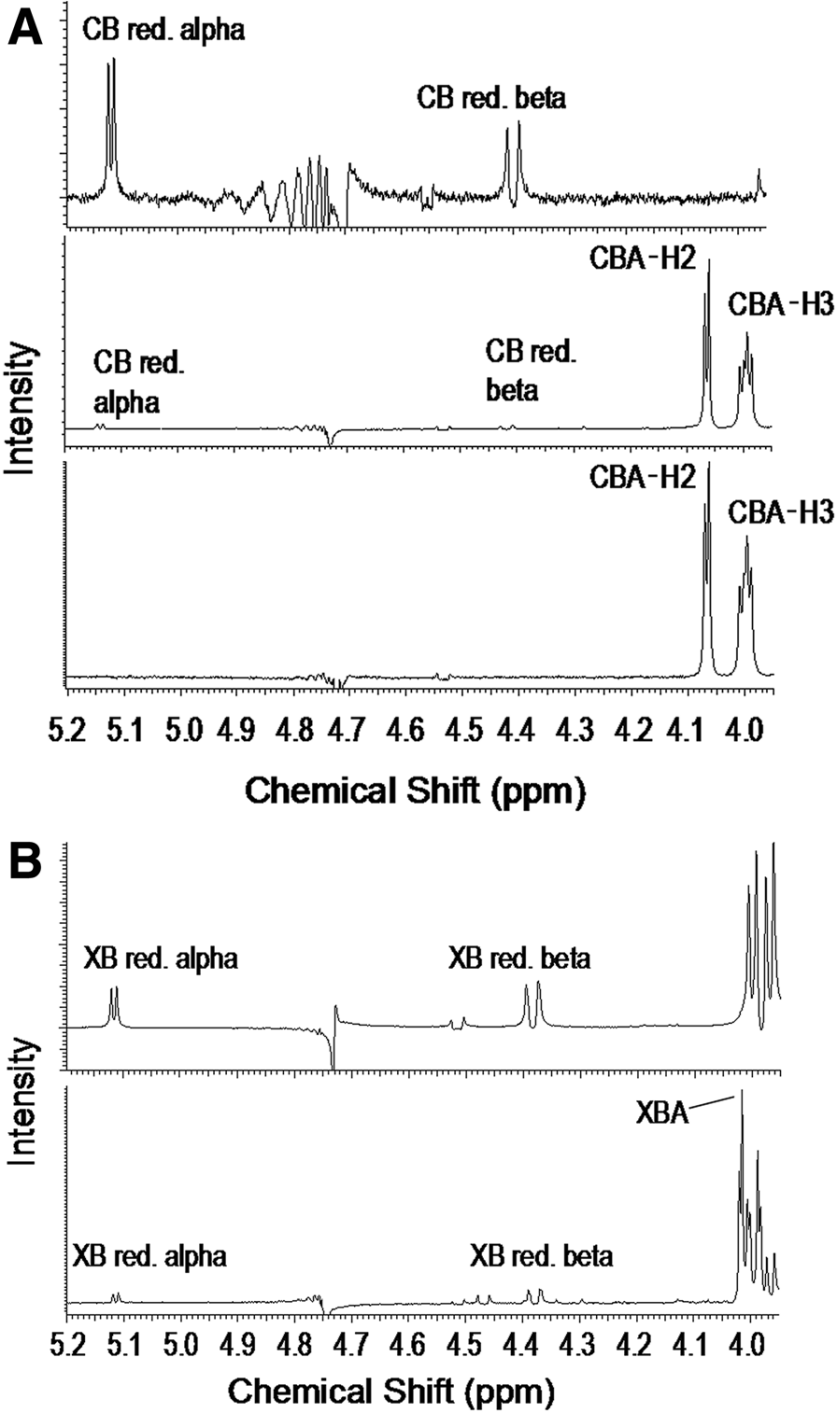
**NMR spectra of cellobiose (A) and xylobiose (B) oxidation. (A)**: From top to bottom are the spectra of cellobiose, cellobiose that was oxidized by GOOX-VN, and cellobiose oxidized by Y300A; CB red. alpha and CB red. beta: H1 signals due to reducing α-glucose and reducing β-glucose units of cellobiose, correspondingly; CBA-H2 and CBA-H3: H2 and H3 signals of the cellobionate molecule. **(B)**: From top to bottom are the spectra of untreated xylobiose and GOOX-VN oxidized xylobiose; XB red. alpha and XB red. beta: H1 signals due to reducing α-xylose and reducing β-xylose units of xylobiose, correspondingly; XBA: Overlapped signals of the xylobionate molecule (H2 and H3 signals were not well separated from other signals). 10 mM cellobiose and 10 mM xylobiose were used in oxidation reactions.

The disappearance of H1 doublet signals from the reducing end of α- and β-glucose units of cellobiose is consistent with oxidation at the anomeric C1 position (Figure 3A) [21]. Similarly, the peak height for the H1 signals from the reducing end of α- and β-xylose units of xylobiose was decreased in oxidized xylobiose samples (Figure 3B). Ring opening at the anomeric position was also revealed by the detection of H2 and H3 signals at 4.05 ppm and 3.95 ppm in case of oxidized cellobiose, and at 4.01 ppm and 3.81 ppm, respectively in case of oxidized xylobiose [21,22]. The signals for the corresponding lactone were not observed probably due to the relatively long oxidation reaction (24 h); similar observations were reported after overnight incubation of *Phanerochaete chrysosporium* CDH with cellobiose [22].

**Figure 4:**
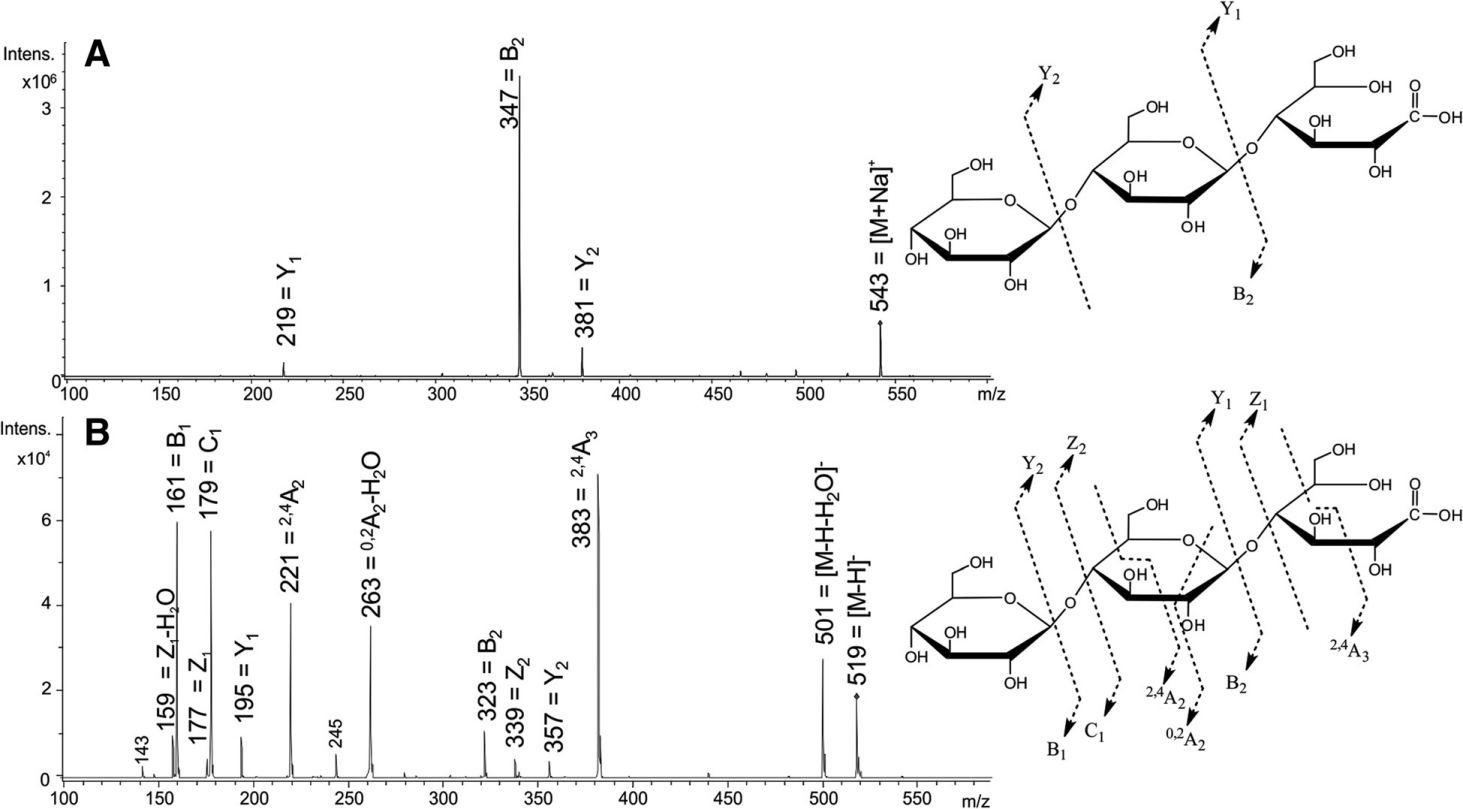
**ESI-MS**/**MS spectra and fragmentation of GOOX-VN oxidized cellotriose. (A)**: MS/MS in the positive ionization (precursor [M+Na]^+^, m/z 543). **(B)**: MS/MS in the negative ionization (precursor [M-H]^-^, m/z 519). Fragment ions were named according to Domon and Costello [33].

ESI-MS/MS analyses also indicated enzymatic oxidation of cellotriose at the anomeric carbon. In the positive ionization mode, the acidic fraction of oxidized cellotriose only produced glycosidic bond cleavage fragments, generating B- and Y-ions (Figure 4A); cross ring cleavage fragmentation was not observed. Since neutral reducing oligosaccharides usually form cross ring cleavage fragments from reducing ends if a sodium cation is present [23,24], oxidation of the anomeric carbon seemed to change the fragmentation behaviour of sodium cationized cellotriose. In the negative mode, B- and C-ions from glycosidic bond cleavage were the most abundant fragment ions (Figure 4B). The molecular masses of Y- and Z-ions increased by 16 Da, compared to the unoxidized control sample in our study (data not shown) or reported in the literature [25], supporting that the oxidation reaction occurred in the reducing glucose. Cross ring cleavage fragmentation was also observed in the negative mode. For instance, a peak at the *m*/*z* ratio of 383 was generated from oxidized cellotriose (m/z 519) by the loss of 136 Da from cross ring cleavage of the oxidized monosaccharide unit, leading to the formation of a ^2,4^A_3_-ion (Figure 4B).

Additional, indirect evidence, from colorimetric assays, for the oxidation at C1 is that no activity was detected on D-glucose derivatives lacking a C1 hydroxyl group, including 1,5-anhydroglucitol (D-glucose with -H instead of -OH at C1) and methyl-β-D-glucopyranoside (D-glucose with -OCH_3_ instead of -OH at C1).

### Reconstructing the recombinant wild-type GOOX

The double substitution (V38A-N388S) was created in GOOX-VN to produce GOOX, the recombinant wild type oxidase of *S*. *strictum* CBS 346.70, while the single N388S substitution was created to generate GOOX-V and investigate the significance of V38.

**Figure 5:**
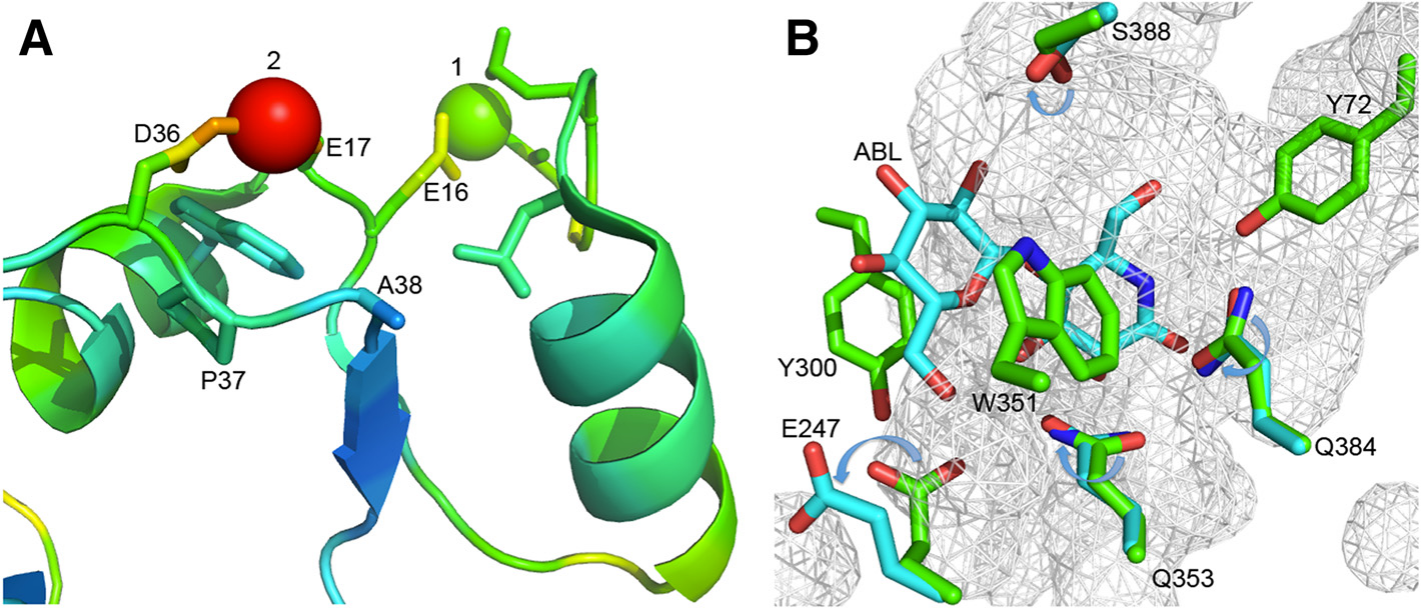
**Structural analysis of GOOX**-**T1. (A)**: Metal binding sites of the FAD-binding domain (coloring by b-factor); A38 is close to the zinc ion 2 bound to D36 and E17. **(B)**: Residues for mutation in relation to the substrate analog, 5-amino-5-deoxy-cellobiono-1,5-lactam (ABL); the movement of residues in the absence of ABL (PDB ID: 1ZR6, green) compared with the presence of ABL (PDB ID: 2AXR, cyan) are indicated by arrows.

The presence of the single A38V mutation increased the catalytic efficiency of GOOX-V on all tested substrates compared to the wild-type enzyme; by contrast, the introduction of both random mutations A38V and S388N (generating GOOX-VN) reduced enzyme activity (Table 2). More specifically, comparisons of GOOX and GOOX-V revealed that the random mutation of A38 to valine did not significantly change the *K*_*m*_ but nearly doubled the *k*_cat_ on all tested substrates. It was initially surprising that a mutation at position 38 affected enzyme activity since this position is close to the protein surface and nearly 30 Å away from the oxidation site. Structural analysis of GOOX-T1 showed that A38 is located on a flexible loop before the β2-sheet and it is close to D36 and E17, which are predicted to coordinate one of the four zinc ions identified in GOOX-T1 crystals grown in the presence of 10 mM ZnSO_4_ (Figure 5) [20]. Since high quality crystals of GOOX-T1 were only formed in the presence of zinc ions, it is possible that divalent ions coordinated by amino acids near A38 participate in stabilizing the protein structure. Notably, the addition of 1 mM ZnCl_2_ slightly increases the specific activity of GOOX-T1, while 1 mM EDTA slightly reduces the specific activity of the enzyme [10]. Since the β2-sheet, together with the β3-sheet and the β4-sheet, forms a P-loop structure that participates in coordination [20], it is conceivable that the A38V substitution affects GOOX activity through an impact on cofactor binding.

**Table 2:** **Kinetic parameters of GOOX**, **GOOX**-**VN** (**A38V**-**S388N**) **and GOOX**-**V** (**A38V**) **on cello**-**oligosaccharides and xylo**-**oligosaccharides**

	**GOOX**			**GOOX-VN (A38V-S388N)**			**GOOX-V (A38V)**		
	***k***_**cat **_**(min**^**-1**^**)**	***K***_**m **_**(mM)**	***k***_**cat**_**/*K***_**m **_**(mM**^**-1 **^**min**^**-1**^**)**	***k***_**cat **_**(min**^**-1**^**)**	***K***_**m **_**(mM)**	***k***_**cat**_**/*K***_**m **_**(mM**^**-1 **^**min**^**-1**^**)**	***k***_**cat **_**(min**^**-1**^**)**	***K***_**m **_**(mM)**	***k***_**cat**_**/*K***_**m **_**(mM**^**-1 **^**min**^**-1**^**)**
Glucose	378 ± 5^*^	15 ± 1	25 ± 2	361 ± 5	15 ± 1	24 ± 2	625 ± 12	12 ± 1	50 ± 5
Cellobiose	420 ± 20	0.04 ± 0.01	9,400 ± 2,000	372 ± 14	0.05 ± 0.01	7,000 ± 1,000	719 ± 8	0.039 ± 0.002	18,400 ± 900
Cellotriose	412 ± 16	0.05 ± 0.01	8,600 ± 1,000	436 ± 14	0.08 ± 0.01	5,600 ± 600	820 ± 50	0.05 ± 0.01	15,000 ± 3,000
Cellotetraose	595 ± 16	0.07 ± 0.01	9,000 ± 800	470 ± 12	0.09 ± 0.01	5,200 ± 500	770 ± 20	0.06 ± 0.01	14,000 ± 2,000
Cellopentaose	498 ± 19	0.06 ± 0.01	8,700 ± 1,100	453 ± 14	0.09 ± 0.01	4,700 ± 400	760 ± 30	0.057 ± 0.007	13,000 ± 2,000
Cellohexaose	430 ± 30	0.08 ± 0.02	5,500 ± 1,200	393 ± 15	0.10 ± 0.01	4,000 ± 400	790 ± 30	0.08 ± 0.01	9,000 ± 1,000
Xylose	444 ± 12	129 ± 8	3.4 ± 0.2	330 ± 8	118 ± 7	2.8 ± 0.2	741 ± 15	118 ± 7	6.3 ± 0.4
Xylobiose	522 ± 8	0.057 ± 0.003	9,100 ± 500	449 ± 5	0.099 ± 0.005	4,500 ± 200	890 ± 20	0.05 ± 0.01	18,000 ± 2,000
Xylotriose	620 ± 14	0.08 ± 0.01	8,200 ± 700	524 ± 10	0.10 ± 0.01	5,100 ± 400	870 ± 30	0.08 ± 0.01	11,000 ± 1,000
Xylotetraose	583 ± 14	0.07 ± 0.01	8,200 ± 800	488 ± 7	0.11 ± 0.01	4,400 ± 300	960 ± 30	0.067 ± 0.007	14,000 ± 1,000
Xylopentaose	623 ± 18	0.05 ± 0.01	11,800 ± 1,400	529 ± 7	0.104 ± 0.005	5,100 ± 200	910 ± 30	0.05 ± 0.01	17,000 ± 2,000
Xylohexaose	516 ± 18	0.06 ± 0.01	8,100 ± 1,200	465 ± 7	0.10 ± 0.01	4,400 ± 300	910 ± 20	0.050 ± 0.004	18,000 ± 2,000
A^3^X	530 ± 13	0.059 ± 0.005	8,900 ± 700	ND	ND	ND	740 ± 14	0.052 ± 0.004	14,000 ± 1,000
A^d2+3^XX	540 ± 11	0.078 ± 0.004	6,900 ± 400	ND	ND	ND	810 ± 20	0.09 ± 0.01	9,000 ± 700
U^4m2^XX	659 ± 9	0.30 ± 0.01	2,190 ± 60	ND	ND	ND	ND	ND	ND

*Data are mean values ± standard errors; 16 nM of enzyme was used in each reaction.

*ND* Not determined.

**Figure 6:**
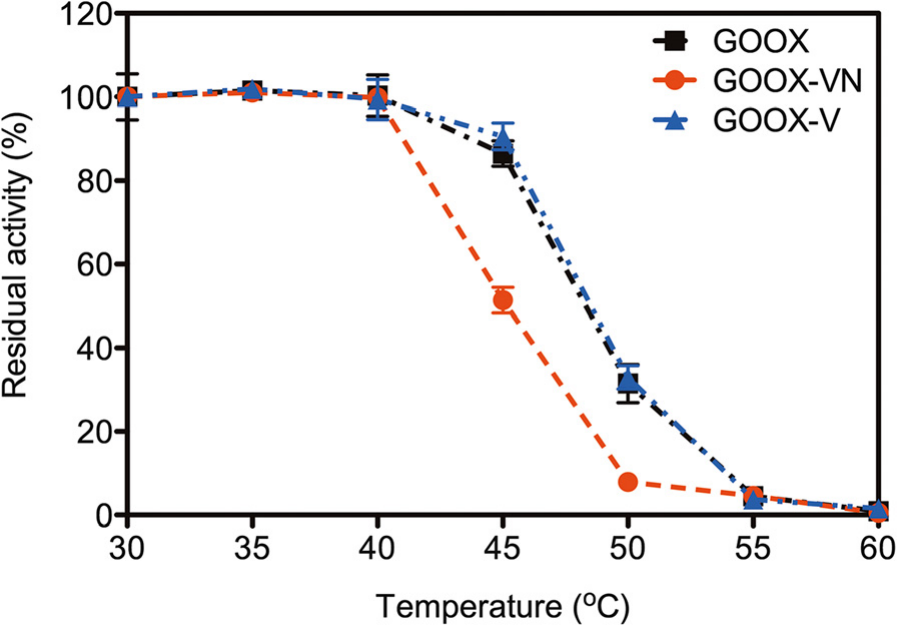
**Thermostability of GOOX**, **GOOX-V and GOOX-VN.** Residual activity on 0.5 mM cellobiose, after a 60-min incubation at different temperatures was measured at 37°C for 15 min at pH 8.0.

S388 is located in the β16-sheet, which forms part of the substrate-binding domain (Figure 5). This position is close to a loop region formed by residues Y390 to N394, which is absent in all of the 30 closest homologs of GOOX (analysis performed at http://consurf.tau.ac.il/). The structure of GOOX-T1 bound by a substrate analogue, 5-amino-5-deoxycellobiono-1,5-lactam (ABL) showed that the side chain of S388 rotates upon ABL binding to form a weak H-bond with G349 [20], which is predicted to stabilize the β16-sheet. In this case, the comparatively large side chain of asparagine could lead to steric destabilization of the protein. This possibility is consistent with the comparatively low thermostability of GOOX-VN compared to GOOX and GOOX-V (Figure 6), which could indirectly alleviate the beneficial affect of the A38V substitution.

### Impact of chain length and sugar composition on GOOX, GOOX-V and GOOX-VN activity

Mass spectrometric analysis of oxidized cello-oligosaccharides from cellobiose to cellopentaose revealed a 16 Da increase in m/z values of the acidic fraction (Additional file 3: Figure S3M-P) compared to the control, unoxidized samples (Additional file 3: Figure S3A-D), confirming that in all cases, the oxidation by GOOX-VN introduced a single oxygen atom to all the oligomeric substrates. The oxidation of different cello-oligosaccharides was efficient, but not complete at the tested concentrations, as can be seen from small amount of unoxidized oligosaccharides detected in the neutral fraction (Additional file 3: Figure S3I-L). Nevertheless, GOOX production of oxidized cello-oligosaccharides might be an efficient way to generate oxidized carbohydrate standards to facilitate the characterisation of the C1-oxidizing enzymes of families AA-9 and AA10.

H_2_O_2_-based colorimetric detection was then used to compare the catalytic efficiency of GOOX, GOOX-VN and GOOX-V. Those analyses confirmed that the catalytic efficiencies of these enzymes are over two orders of magnitude higher on oligomeric substrates compared to corresponding monosaccharides (Table 2). Notably, however, among the oligomeric substrates, catalytic efficiencies with cello-oligosaccharides and xylo-oligosaccharides decreased slightly with increasing degree of polymerization, mainly due to increasing *K*_*m*_ values (Table 2). This observation is consistent with earlier predictions of two binding subsites in GOOX enzymes [20].

GOOX-VN, GOOX-V and GOOX effectively oxidized xylo-oligosaccharides as well as cello-oligosaccharides, and GOOX displayed even higher catalytic efficiency on xylopentaose and xylohexaose than on the corresponding cello-oligosaccharides (Table 2). The catalytic efficiency of GOOX and GOOX-V on a substituted xylo-oligosaccharide (A^3^X) and a branched xylo-oligosaccharide (A^d2+3^XX) was comparable to unsubstituted and unbranched substrates, indicating that these sugar substitutions do not interfere with GOOX activity (Table 2). However, the catalytic efficiency of GOOX on U^4m2^XX was significantly reduced due to high *K*_m_ values (Table 2), suggesting comparatively poor binding of anionic oligosaccharides by GOOX enzymes. Regardless of a similarly low activity on xylose and two other monosaccharides: N-acetylglucosamine and galactose [7], GOOX and GOOX-VN were not active on chitobiose, chitotriose, and galactobiose, suggesting that substrate interaction at the -2 binding subsite plays an important role for substrate specificity.

### Key residues involved in substrate binding

Heuts et al. [26] reveal that by substituting one residue in the substrate recognition site of *F*. *graminearum* chito-oligosaccharide oxidase, the enzyme gains activity on gluco-oligosaccharides. Accordingly, detailed site-directed mutagenesis was performed to study the contribution of substrate-binding site residues on the substrate specificity and catalytic efficiency of GOOX. Close examination of GOOX-T1 and GOOX-VN identified five amino acids in the substrate-binding domain that were targeted for single mutation in GOOX-VN. Of the chosen residues, Y72 and Q384 are at the -1 binding subsite, E247 and W351 are at the -2 binding subsite and Q353 is between the two binding subsites (Figure 5). While most substitutions were to alanine, some were mutated to related residues to evaluate the impact of amino acid size on enzyme activity. Since the catalytic efficiency of GOOX-VN was not dramatically affected by oligosaccharide length (Table 2), oligosaccharides with up to 3 sugar units instead of 6 units were used to obtain kinetic parameters for the mutant enzymes.

In all cases, *K*_m_ values for the mutant enzymes were higher than GOOX-VN, consistent with the role of each substituted residue in substrate binding (Table 3). For instance, the removal of either the hydroxyl group (Y72F) or the complete side chain (Y72A) of an amino acid contributing to the -1 subsite increased the *K*_m_ of corresponding enzymes, particularly on xylo-oligosaccharides (Table 3). Notably, Y72 interacts with the endocyclic O^5^ and can hydrogen-bond with OH^6^ of the cellobionolactone analog [20]. Huang et al. [14] observed that the H70A substitution in GOOX-T1, which removes a covalent linkage to the FAD cofactor, also increases *K*_m_ values. Given the close positioning of H70 and Y72, higher *K*_*m*_ values in H70A mutants might result from indirect effects on Y72 positioning.

**Table 3:** **Kinetics of GOOX**-**VN mutant enzymes on cello**- **and xylo**-**oligosaccharides**

**Substrate**	**Parameter**	**GOOX-VN**	**Y72F**	**Y72A**	**E247A**	**W351A**	**Q353N**	**Q353A**	**Q384N**	**Q384A**
Glucose	*k*_cat_ (min^-1^)	361 ± 5	-	-	472 ± 6	(1,280 ± 70)^*^	-	-	(713 ± 14)	-
	*K*_m_ (mM)	15 ± 1	-	-	62 ± 2	(890 ± 60)	-	-	(325 ± 10)	-
	*k*_cat_/*K*_m_ (mM^-1^ min^-1^)	24 ± 2	-	-	7.7 ± 0.3	(1.4 ± 0.1)	-	-	(2.2 ± 0.1)	-
Cellobiose	*k*_cat_ (min^-1^)	372 ± 14	497 ± 7	644 ± 8	480 ± 14	1,040 ± 30	117 ± 2	16 ± 1	916 ± 13	210 ± 4
	*K*_m_ (mM)	0.05 ± 0.01	1.3 ± 0.1	2.5 ± 0.1	0.17 ± 0.02	13 ± 1	11.3 ± 0.4	1.1 ± 0.2	0.63 ± 0.03	8.8 ± 0.3
	*k*_cat_/*K*_m_ (mM^-1^ min^-1^)	7,000 ± 1,000	370 ± 20	250 ± 12	2,800 ± 300	79 ± 5	10.3 ± 0.4	15 ± 3	1,450 ± 80	24 ± 1
Cellotriose	*k*_cat_ (min^-1^)	436 ± 14	533 ± 5	695 ± 6	465 ± 17	1,057 ± 15	124 ± 2	20.0 ± 0.3	1,032 ± 12	245 ± 3
	*K*_m_ (mM)	0.08 ± 0.01	0.62 ± 0.02	1.17 ± 0.03	0.11 ± 0.01	6.6 ± 0.2	6.2 ± 0.2	0.57 ± 0.03	0.20 ± 0.01	4.6 ± 0.1
	*k*_cat_/*K*_m_ (mM^-1^ min^-1^)	5,600 ± 600	860 ± 30	596 ± 15	4,300 ± 500	160 ± 5	20 ± 1	35 ± 2	5,000 ± 200	54 ± 2
Xylose	*k*_cat_ (min^-1^)	330 ± 8	-	-	189 ± 5	-	-	-	-	-
	*K*_m_ (mM)	118 ± 7	-	-	167 ± 11	-	-	-	-	-
	*k*_cat_/*K*_m_ (mM^-1^ min^-1^)	2.8 ± 0.2	-	-	1.1 ± 0.1	-	-	-	-	-
Xylobiose	*k*_cat_ (min^-1^)	449 ± 5	(446 ± 9)	764 ± 17	536 ± 6	(670 ± 50)	-	-	(332 ± 11)	(115 ± 7)
	*K*_m_ (mM)	0.099 ± 0.005	(10.3 ± 0.3)	7.0 ± 0.3	0.43 ± 0.02	(48 ± 4)	-	-	(11 ± 1)	(23 ± 2)
	*k*_cat_/*K*_m_ (mM^-1^ min^-1^)	4,500 ± 200	(43 ± 2)	109 ± 5	1,250 ± 60	(14 ± 2)	-	-	(30 ± 2)	(5.0 ± 0.5)
Xylotriose	*k*_cat_ (min^-1^)	524 ± 10	(590 ± 30)	920 ± 40	620 ± 20	(1,500 ± 300)	-	-	(260 ± 30)	(270 ± 30)
	*K*_m_ (mM)	0.10 ± 0.01	(11 ± 1)	6.1 ± 0.5	0.34 ± 0.04	(100 ± 20)	-	-	(10 ± 2)	(41 ± 6)
	*k*_cat_/*K*_m_ (mM^-1^ min^-1^)	5,100 ± 400	(54 ± 6)	150 ± 14	1,800 ± 200	(15 ± 5)	-	-	(27 ± 5)	(7 ± 1)

^*^Estimated data are in parenthesis since *K*_m_ values exceeded the tested substrate concentrations.

-Activity was not detected.

The impact of amino acid substitutions on catalytic rates was more varied. For instance, *k*_cat_ values of W351A on all cello-oligosaccharides were nearly three times higher than corresponding values for GOOX-VN. Similarly, the *k*_cat_ value of W351A on xylotriose was nearly triple that of GOOX-VN, even though activity on xylose was not detectable (Table 3). Q384A lost activity on all substrates; however, the presence of asparagine at this position could partially recover that loss, particularly on cello-oligosaccharides (Table 3). Interestingly, the *k*_cat_ of Q384N on glucose and cello-oligosaccharides was nearly doubled compared to GOOX-VN. The distance between the O^1^ atom of the β anomer to the O^η^ of the catalytic base Y429 and to the N^ϵ2^ of Q384 is approximately 3.1 Å and 3.7 Å, respectively [20]. It is possible that the shorter asparagine side chain could improve the positioning of C6 substrates relative to the catalytic base, thereby increasing *k*_cat_. Accordingly, the Q384N variant might be particularly useful when wanting to selectively oxidize glucose and cello-oligosaccharides in mixtures containing xylo-oligosaccharides.

The mutation of Q353 to alanine eliminated enzymatic activity, which was not recovered by replacing the alanine by asparagine (Table 3). Q353 forms two hydrogen bonds with the ABL substrate analog: one with the OH^3^ at the -1 subsite and the other with OH^6^ at the -2 subsite, which is the only direct protein-carbohydrate hydrogen bond in the -2 subsite [20]. Structural analyses of GOOX-T1 bound by ABL also show that E247 shifts away from the oxidation site, up to 4.6 Å from the unbound reference structure (Figure 5). The E247A variant displayed slightly reduced catalytic efficiency on all tested substrates, generally resulting from increased *K*_m_ values (Table 3). This result suggests that the predicted side chain movement at this position is not crucial for enzyme activity.

### Alleviating substrate inhibition through mutagenesis of aromatic, subsite residues

**Figure 7:**
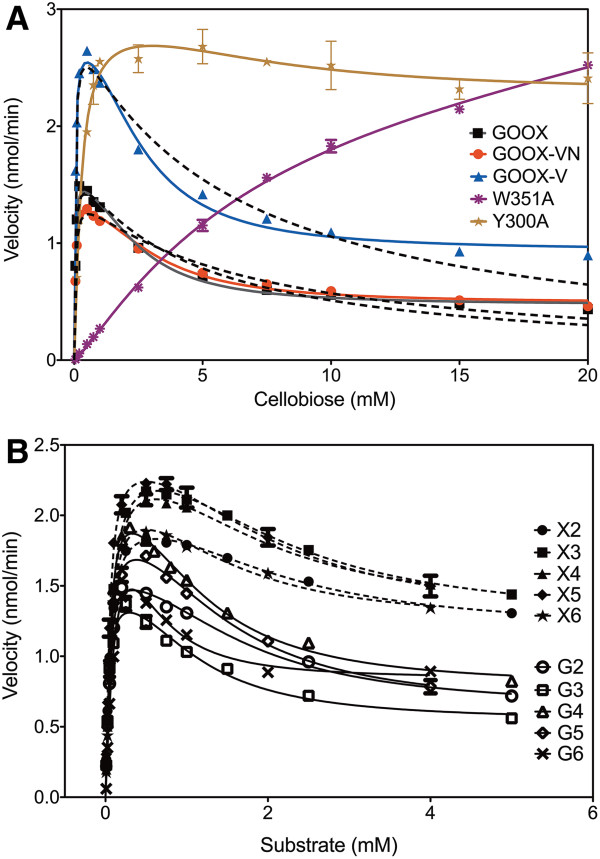
**Substrate inhibition. (A)**: Substrate inhibition models of GOOX, GOOX-V and GOOX-VN; dashed lines indicate substrate inhibition curves fitted with eq. 1 (uncompetitive substrate inhibition model); solid lines indicate inhibition curves fitted with eq. 2 (a modified Hill equation [27]). **(B)**: Inhibition of xylo-oligosaccharides (dashed lines) and cello-oligosaccharides (solid lines) on GOOX activity; curves fitted using eq. 2; X2 to X6 - xylobiose to xylohexaose, correspondingly and G2 to G6 - cellobiose to cellohexaose, correspondingly.

The activity of GOOX and its two mutant enzymes GOOX-VN and GOOX-V was reduced at comparatively high oligosaccharide concentrations, consistent with substrate inhibition. A modified Hill model (Equation 2) [27] described the activity data better than the conventional uncompetitive substrate inhibition model (Equation 1) (Figure 7A); therefore, inhibition kinetics parameters were calculated using Equation 2 (Table 4). *V*_i_/*V*_max_ ratios were also calculated since residual activities were measured for all substrates and substrate concentrations tested, although to different levels. Most notably, *V*_i_/*V*_max_ values for cello-oligosaccharides were lower than for xylo-oligosaccharides, indicating that the activity of all three GOOX variants was inhibited more severely by cello-oligosaccharides than xylo-oligosaccharides (Figure 7B). Consistent with corresponding *K*_m_ values (Table 2), *V*_i_/*V*_max_ values slightly increased with increasing cello-oligosaccharide chain length (Table 4). These inhibition data also indicate that GOOX can be a useful tool for glucose as well as xylose oxidation at very high substrate concentrations.

**Table 4:** Substrate inhibition of GOOX and mutant variants

**Substrate**	**GOOX**			**GOOX-VN**			**GOOX-V**			**Y300A**	**W351A**
	***V***_**i**_**/*V***_**max **_**(%)**	***K***_**i **_(mM)^*^	***n***^H**^	***V***_**i**_**/*V***_**max **_**(%)**	***K***_**i **_**(mM)**	***n***^**H**^	***V***_**i**_**/*V***_**max **_**(%)**	***K***_**i **_**(mM)**	***n***^**H**^		
Glucose	NI			NI			NI			NI	NI
Cellobiose	32	2.51 ± 0.18	2.2	38	3.2 ± 0.2	1.8	35	2.7 ± 0.2	1.3	NI	NI
Cellotriose	34	1.33 ± 0.13	1.4	42	1.9 ± 0.2	1.3	37	1.56 ± 0.13	1.9	NI	NI
Cellotetraose	34	1.49 ± 0.10	1.8	42	2.20 ± 0.16	1.5	36	1.61 ± 0.12	1.6	NI	NI
Cellopentaose	35	1.53 ± 0.13	1.4	53	1.66 ± 0.18	1.3	43	1.36 ± 0.11	1.4	NI	NI
Cellohexaose	44	0.62 ± 0.09	1.3	54	0.81 ± 0.10	1.1	44	0.83 ± 0.07	1.2	NI	NI
Xylose	ID			ID			ID			NI	NI
Xylobiose	51	3.1 ± 0.2	1.2	70	5.0 ± 0.6	1.1	57	3.0 ± 0.2	1.5	NI	NI
Xylotriose	45	2.99 ± 0.19	1.3	75	3.6 ± 0.4	1.2	49	3.0 ± 0.3	1.5	NI	NI
Xylotetraose	57	1.94 ± 0.16	1.3	NI			62	1.82 ± 0.15	1.3	NI	NI
Xylopentaose	53	2.0 ± 0.3	1.4	NI			56	2.0 ± 0.4	1.4	ND	NI
Xylohexaose	61	1.6 ± 0.2	1.5	NI			54	2.2 ± 0.3	1.3	NI	NI

^*^Substrate inhibition kinetics derived from eq. 2. ^**^Hill coefficient.

^-^Values could not be calculated due to no detectable activity.

^NI^No inhibition detected at tested substrate concentrations; ^ID^Insufficient data to calculate inhibition kinetics; however inhibition was observed at xylose concentrations greater than 800 mM; ^ND^Not determined.

Structural analyses suggest that non-productive substrate binding at high substrate concentrations could be stabilized through stacking interactions with amino acid residues above the Y300 and W351 -2 subsite (Additional file 4: Figure S4). This possibility of non-productive cooperative binding at the -2 subsite is also consistent with alleviated inhibition observed upon Y300A [7] and W351A substitution (Table 4 and Figure 7A). Moreover, ^1^H NMR analyses were consistent with less inhibition of the Y300A enzyme by cellobiose than GOOX-VN (Figure 3A). Specifically, the H1 signals due to reducing α-glucose and reducing β-glucose units of cellobiose completely disappeared when 10 mM cellobiose was oxidized by Y300A while their residual signals were detected when the same cellobiose concentration was oxidized by GOOX-VN. These analyses suggest that W351A and Y300A mutant enzymes might be ideal candidates for oxidizing otherwise inhibitory oligosaccharides when present at high substrate concentrations.

## Conclusions

The double mutation of V38A-N388S in GOOX-VN to regenerate the recombinant wild type oxidase of *S*. *strictum* CBS 346.70 confirmed that the reverse mutations do not explain the difference in substrate preference between GOOX-VN and GOOX-T1. The current analysis also further highlights that GOOX enzymes characterized to date are not specific to glucose-based substrates, and instead show broad substrate specificity on a number of oligosaccharides including cello- and xylo-oligosaccharides, as well as substituted and branched xylo-oligosaccharides. The substrate promiscuity of GOOX, along with variants with higher catalytic activity and lower substrate inhibition, broadens its applications in biomass processing at high polysaccharide and oligosaccharide concentrations.

## Methods

### Materials

*Sarocladium strictum* type strain CBS 346.70 was obtained from the American Type Culture Collection (ATCC) No.34717. Glucose, xylose, and cellobiose were purchased from Sigma (St. Louis, USA), while other cello-oligosaccharides as well as xylo-oligosaccharides, chito-oligosaccharides and galactobiose were purchased from Megazyme (Megazyme International, Ireland). Substituted xylo-oligosaccharides including α-L-arabinofuranosyl-(1→3)-β-D-xylopyranosyl-(1→4)-D-xylose (A^3^X), α-L-arabinofuranosyl-(1→2)-[α-L-arabinofuranosyl-(1→3)]-β-D-xylopyranosyl-(1→4)-β-D-xylopyranosyl-(1→4)-D-xylose (A^d2+3^XX) and 4-*O*-methyl-α-D-glucopyranosyl uronic acid-(1→2)-β-D-xylopyranosyl- (1→4)-β-D-xylopyranosyl-(1→4)-D-xylose (U^4m2^XX) were prepared as previously described [28-30].

### Site-directed mutagenesis

The QuikChange kit (Agilent Technologies, USA) and ten primer pairs (Additional file 5: Table S1) were used to separately introduce ten amino acid substitutions to GOOX-VN. The GOOX-VN gene from *S*. *strictum* CBS 346.70 that was previously cloned into the pPICZαA expression vector [7] was used as the template for site-directed mutagenesis. Expression plasmids containing the mutated gene were sequenced at the Centre for Applied Genomics (TCAG, the Hospital for Sick Children).

### Recombinant protein expression

Mutated plasmids were transformed into *Pichia pastoris* strains according to the manufacturer’s instructions (Life Technologies, USA). The transformants were screened for protein expression by immuno-colony blot as previously described [7] as well as using an overlay activity assay. Briefly, approximately 10 mL of the overlay mixture (0.3% agarose, 2% cellobiose, 2 mM phenol, 0.4 mM 4-aminoantipyrine (4-AA) and 15 U/mL horseradish peroxidase in 50 mM Tris–HCl pH 8.0) were applied over *P*. *pastoris* colonies that had been induced for 3 days by daily addition of 0.5% methanol. Following 30 min to 60 min of incubation at 37°C, transformants that expressed active forms of the recombinant enzyme were identified by the formation of a pink halo around the colony. Positive transformants were grown at 30°C and 250 rpm for 5 days, and 0.5% methanol was added every 24 h to induce recombinant protein expression.

Culture supernatants were collected by centrifugation at 5,000 g for 10 min, and then concentrated to approximately 15 mL using Jumbosep™ centrifugal concentrator units (Pall Corporation, USA) before being passed through a HiTrap™ desalting column (GE Healthcare, UK) using a BioLogic Duoflow FPLC system (Bio-Rad Laboratories, USA). The protein fractions were loaded onto a GE HisTrap™ column (GE Healthcare, UK), washed with the washing buffer (50 mM NaH_2_PO_4_, 300 mM NaCl, 20 mM imidazole, pH 8.0) and then eluted using the elution buffer, which is the same as the washing buffer but with 250 mM imidazole. Eluted fractions were replaced by 50 mM Tris–HCl (pH 8.0) using Vivaspin 20 concentration units (Sartorius, Germany).

### Confirmation of protein purity and identity

Protein concentrations were measured using the Bradford method (Bio-Rad Laboratories, USA) and confirmed using SDS-PAGE densitometry, where the band density of GOOX and the BSA reference protein were determined using ImageJ (http://rsbweb.nih.gov/ij/). Retention of the FAD cofactor in mutant enzymes was assessed by verifying sample absorbance at 350–700 nm using a Varian Cary 50 Bio UV–VIS spectrophotometer (Agilent Technologies, USA). The presence of the FAD cofactor in intact protein samples was further confirmed by running the enzyme samples using SDS-PAGE and incubating the gel in 10% acetic acid for 10 min before visualization of fluorescent bands under a hand-held Mineralight® UV lamp (UVP, USA). A second, identical SDS-PAGE gel was treated with performic acid before the acetic acid treatment to check for increase in fluorescence intensity [19] (note: extra caution is required when handling performic acid).

To confirm the introduction of single amino acid substitutions, protein samples were exchanged to MilliQ water using 10 kDa Amicon filter units (EMD Millipore, USA), and then 2000 pmol of each protein were processed using a Waters Pico-Tag System to evaluate total amino acid composition (Advanced Protein Technology Centre, Hospital for Sick Children, Toronto, Canada). Protein samples were also digested with modified sequencing-grade trypsin (Promega, USA) and peptide sequences were obtained by tandem mass spectrometry using an LTQ-XL™ mass spectrometer (Thermo Fisher Scientific, USA).

### Enzymatic kinetics and thermostability

A 96-well chromogenic assay was used to measure hydrogen peroxide production [7,10]. Briefly, the production of H_2_O_2_ was coupled to the oxidation of 4-AA by horseradish peroxidase and measured continuously at 500 nm and 37°C for 15 min. To determine specific activity, 16 nM of enzyme was assayed with 0.5 mM oligosaccharides. Kinetic parameters were determined by using 16 nM of enzyme and a range of substrate concentrations: 0.05 - 300 mM glucose, 0.05 - 1200 mM xylose, 0.05 - 20 mM cellobiose, 0.01 - 10 mM cellotriose, xylobiose, and xylotriose, 0.01 - 4 mM of longer cello- and xylo-oligosaccharides, 0.01 - 1 mM A^3^X and A^d2+3^XX, and 0.04 - 0.4 mM U^4m2^XX. At least eight substrate concentrations in triplicates were assayed for each substrate, and then kinetic parameters were calculated using the Michaelis–Menten equation of GraphPad Prism5 software (GraphPad Software, USA). Substrate inhibition kinetics were calculated using a conventional substrate inhibition equation (Equation 1) and a modified Hill equation (Equation 2) [27]:

(1) $$v=\frac{V_{\max}*\left[S\right]}{K_{m}+\left[S\right]+\frac{{\left[S\right]}^{2}}{K_{i}}}$$

(2) $$v=\frac{V_{\max}+V_{i}\left(\frac{{\left[S\right]}^{2}}{K_{i}^{2}}\right)}{1+\frac{K_{s}^{n_{H}}}{{\left[S\right]}^{n_{H}}}+\frac{{\left[S\right]}^{2}}{K_{i}^{2}}}$$

Where, *V*_i_ is the reaction velocity in the presence of inhibition and *n*_H_ is the Hill coefficient.

To determine the temperature stabilities of enzyme variants, 16 nM of each enzyme was incubated for 1 h at temperatures between 30 and 60°C, and residual activities were measured using the conventional 4-AA chromogenic assay and 0.5 mM cellobiose.

### Mass spectrometric analysis of oxidized products

Reaction mixtures containing 1 mM of cello-oligosaccharides, from cellobiose to cellohexaose, and 160 nM GOOX-VN or GOOX-Y300A, in 50 mM Tris HCl (pH 8.0) were incubated overnight at 37°C. To characterize oxidized products, 100 μL of each reaction mixture were diluted in 900 μL of MilliQ-water, and diluted samples were purified and fractionated to neutral and acidic oligosaccharides using a Hypersep porous graphitized carbon column (Thermo Scientific, MA, USA), following the protocols of Packer et al. [31] and Chong et al. [32] with modifications. Neutral sugars were eluted using 40% acetonitrile, and mixture of 50% acetonitrile and 0.05% TFA were used to elute acidic sugars. Collected fractions were dried with nitrogen gas for 20 min and then freeze-dried overnight.

Mass spectrometric analyses were performed using an Agilent XCT Plus model ion trap mass spectrometer (Agilent Technologies, Waldbronn, Germany) equipped with an electrospray source. For ESI-MS and ESI-MS/MS analyses, freeze dried samples were dissolved in 20 μL of MilliQ-water, and 6 μL of each sample was diluted in 100 μL of methanol–water-formic acid solvent (50:49:1 (v:v:v)). Sample solutions were introduced into the ES source at a flow rate of 5 μL/min via a syringe pump. The drying gas temperature was set to 325°C; drying gas flow was 4 L/min; the nebulizer pressure was 15 psi, and the ES capillary voltage was set to 3164 V. Ions were collected in the m/z range of 50 to 1000. ESI-MS/MS analyses were performed in both positive and negative ionization modes. Fragmentation amplitude was set to 0.60 V in the positive mode and 0.80 V in the negative mode, and the precursor ion isolation width was set to 1.0 m/z and 1.5 m/z, respectively.

### NMR analysis of oxidized products

Reaction mixtures containing 10 mM cellobiose or 10 mM xylobiose, and 160 nM GOOX-VN or GOOX-Y300A, in 50 mM Tris HCl (pH 8.0) were incubated overnight at 37°C. Oxidized products were analyzed by proton nuclear magnetic resonance (^1^H NMR) using a Bruker 400 MHz NMR Spectrometer (Bruker Ultrashield 400 Plus, USA). Samples were measured directly in the reaction solvent with water suppression using 10% deuterium oxide as a co-solvent for deuterium lock. The peaks were identified using the estimation program of ChemBioDrawUltra 12.0 (CambridgeSoft).

## Abbreviations

4-AA: 4-aminoantipyrine; AA: auxiliary activities; ABL: 5-amino-5-deoxy-cellobiono-1,5-lactam; CDH: cellobiose dehydrogenases; FAD: flavin adenine dinucleotide; GOOX: gluco-oligosaccharide oxidase; A3X: α-L-arabinofuranosyl-(1→3)-β-D-xylopyranosyl-(1→4)-D-xylose; Ad2+3XX: α-L-arabinofuranosyl-(1→2)-[α-L-arabinofuranosyl-(1→3)]-β-D-xylopyranosyl-(1→4)-β-D-xylopyranosyl-(1→4)-D-xylose; U4m2XX: 4-*O*-methyl-α-D-glucopyranosyl uronic acid-(1→2)-β-D-xylopyranosyl-(1→4)-β-D-xylopyranosyl-(1→4)-D-xylose.

## Competing interests

The authors declare there are no competing interests.

## Authors’ contributions

TVV, JS, MT and ERM designed the experiments. TVV, AHV, MF and MJ performed the experiments and analyzed the data. All authors discussed the results and implications and commented on the manuscript at all stages. All authors read and approved the final manuscript.

## Supplementary Material

Additional file 1: Figure S1 Protein sequence alignment of GOOX-T1, GOOX and GOOX-VN. The protein sequence of GOOX-T1 from *S*. *strictum* T1 was aligned with those of GOOX and GOOX-VN from *S*. *strictum* CBS 346.70. The positions of amino acid differences are numbered while the amino acid substitutions created in GOOX-VN for the current study are indicated by rectangles. Amino acid substitutions introduced to re-construct GOOX from GOOX-VN are indicated by an asterisk. Click here for file

Additional file 2: Figure S2 SDS-PAGE analysis of purified protein preparations. SDS-PAGE was performed using a 12% polyacrylamide gel, which was then stained with Coomassie Brilliant Blue R-250. PageRuler™ Plus prestained protein ladder (Fermentas) was used. Click here for file

Additional file 3: Figure S3 Positive ion ESI-MS spectra of four cello-oligosaccharide samples before and after oxidation. Samples were separated to neutral and acidic fractions prior analysis. G2: Cellobiose; G3: Cellotriose; G4: Cellotetraose; G5: Cellopentaose. (A)-(H): Unoxidized cello-oligosaccharide samples; (I)-(P): GOOX-VN oxidized cello-oligosaccharide samples; (A)-(D) and (I)-(L): Neutral fractions: (E)-(H) and (M)-(P): Acidic fractions. Na: Sodium, K: Potassium and H: Proton adducts, respectively. Click here for file

Additional file 4: Figure S4 A potential non-productive binding subsite. (Left): A potential binding pocket (yellow eclipse) above Y300 and W351, as seen in the GOOX-T1 structure with the presence of ABL (PDB ID: 2AXR). (Right): A 45°-rotated view, the distance between two stacking residues is 8.1 Å. Click here for file

Additional file 5: Table S1 The sequences of forward oligonucleotide primers used for site-directed mutagenesis. Click here for file
