# Supplementary figures and images for: Xylo- and cello-oligosaccharide oxidation by gluco-oligosaccharide oxidase from Sarocladium strictum and variants with reduced substrate inhibition
Source: Biotechnol Biofuels. 2013 Oct 12;6:148. doi: 10.1186/1754-6834-6-148 (PMC4015748; doi:10.1186/1754-6834-6-148)

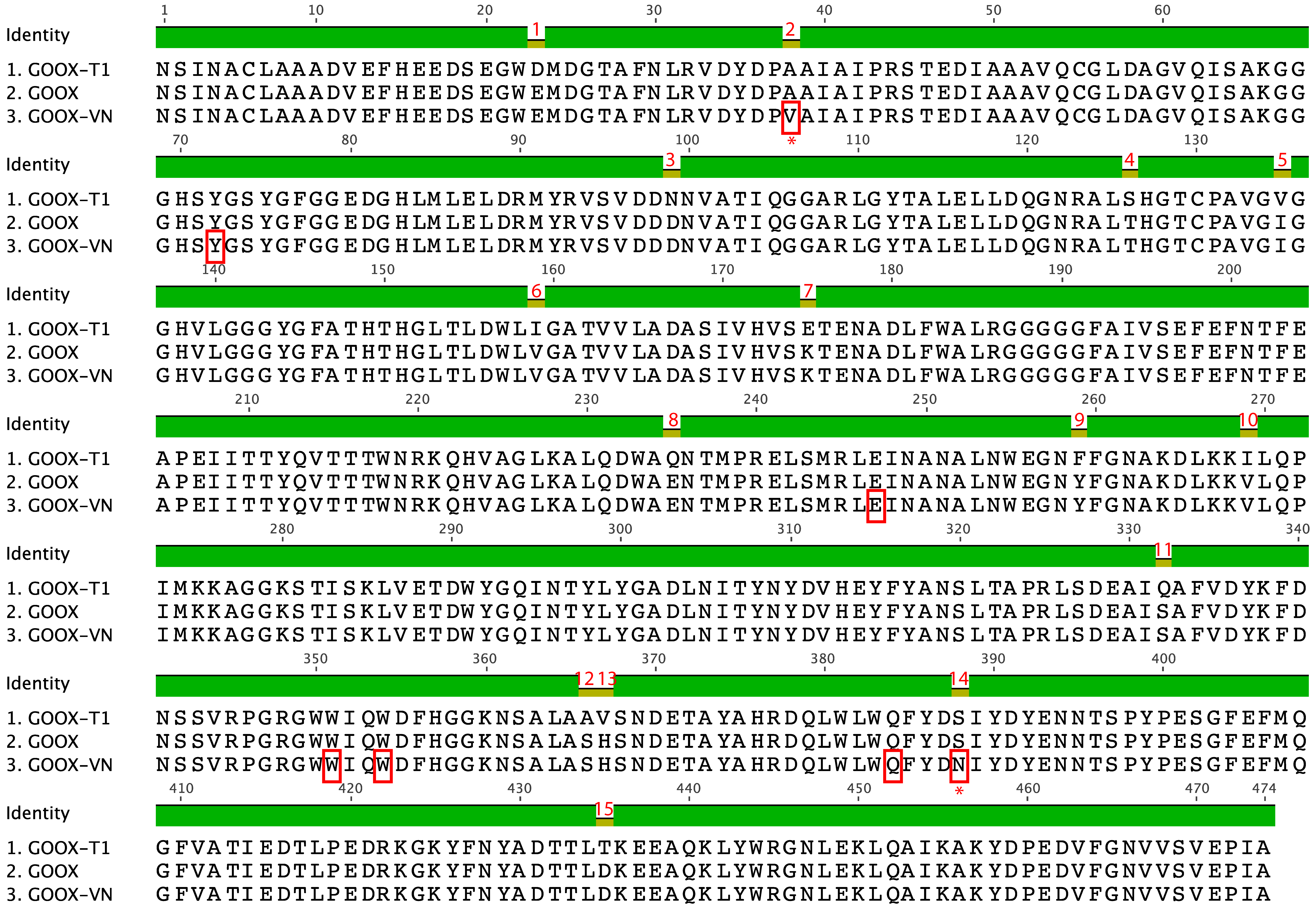

Supplement: Additional file 1: Figure S1 — Protein sequence alignment of GOOX-T1, GOOX and GOOX-VN. The protein sequence of GOOX-T1 from S. strictum T1 was aligned with those of GOOX and GOOX-VN from S. strictum CBS 346.70. The positions of amino acid differences are numbered while the amino acid substitutions created in GOOX-VN for the current study are indicated by rectangles. Amino acid substitutions introduced to re-construct GOOX from GOOX-VN are indicated by an asterisk. [file 1754-6834-6-148-S1.tiff]

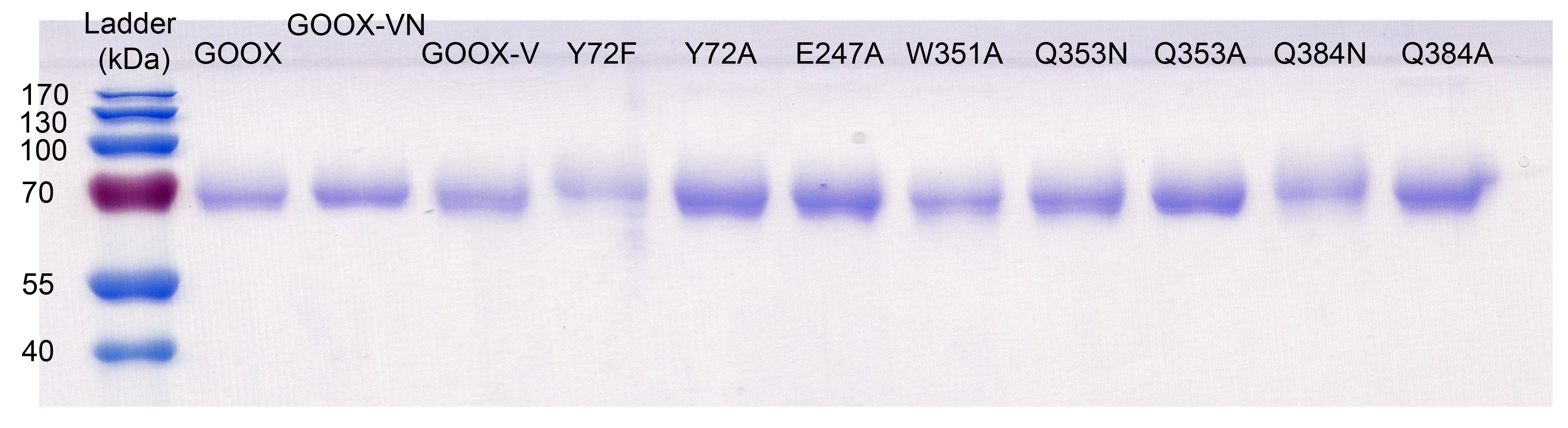

Supplement: Additional file 2: Figure S2 — SDS-PAGE analysis of purified protein preparations. SDS-PAGE was performed using a 12% polyacrylamide gel, which was then stained with Coomassie Brilliant Blue R-250. PageRuler™ Plus prestained protein ladder (Fermentas) was used. [file 1754-6834-6-148-S2.tiff]

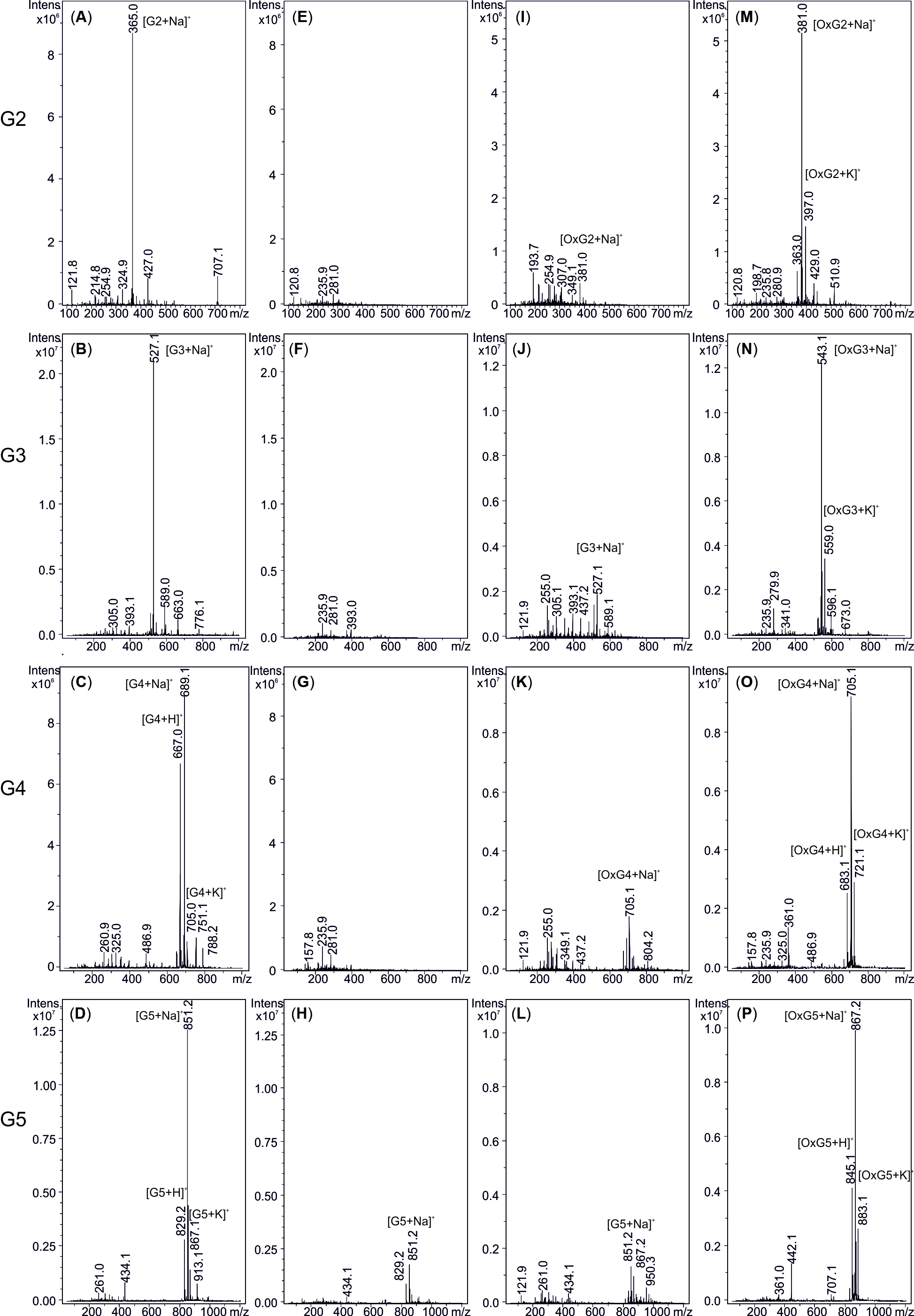

Supplement: Additional file 3: Figure S3 — Positive ion ESI-MS spectra of four cello-oligosaccharide samples before and after oxidation. Samples were separated to neutral and acidic fractions prior analysis. G2: Cellobiose; G3: Cellotriose; G4: Cellotetraose; G5: Cellopentaose. (A)-(H): Unoxidized cello-oligosaccharide samples; (I)-(P): GOOX-VN oxidized cello-oligosaccharide samples; (A)-(D) and (I)-(L): Neutral fractions: (E)-(H) and (M)-(P): Acidic fractions. Na: Sodium, K: Potassium and H: Proton adducts, respectively. [file 1754-6834-6-148-S3.tiff]

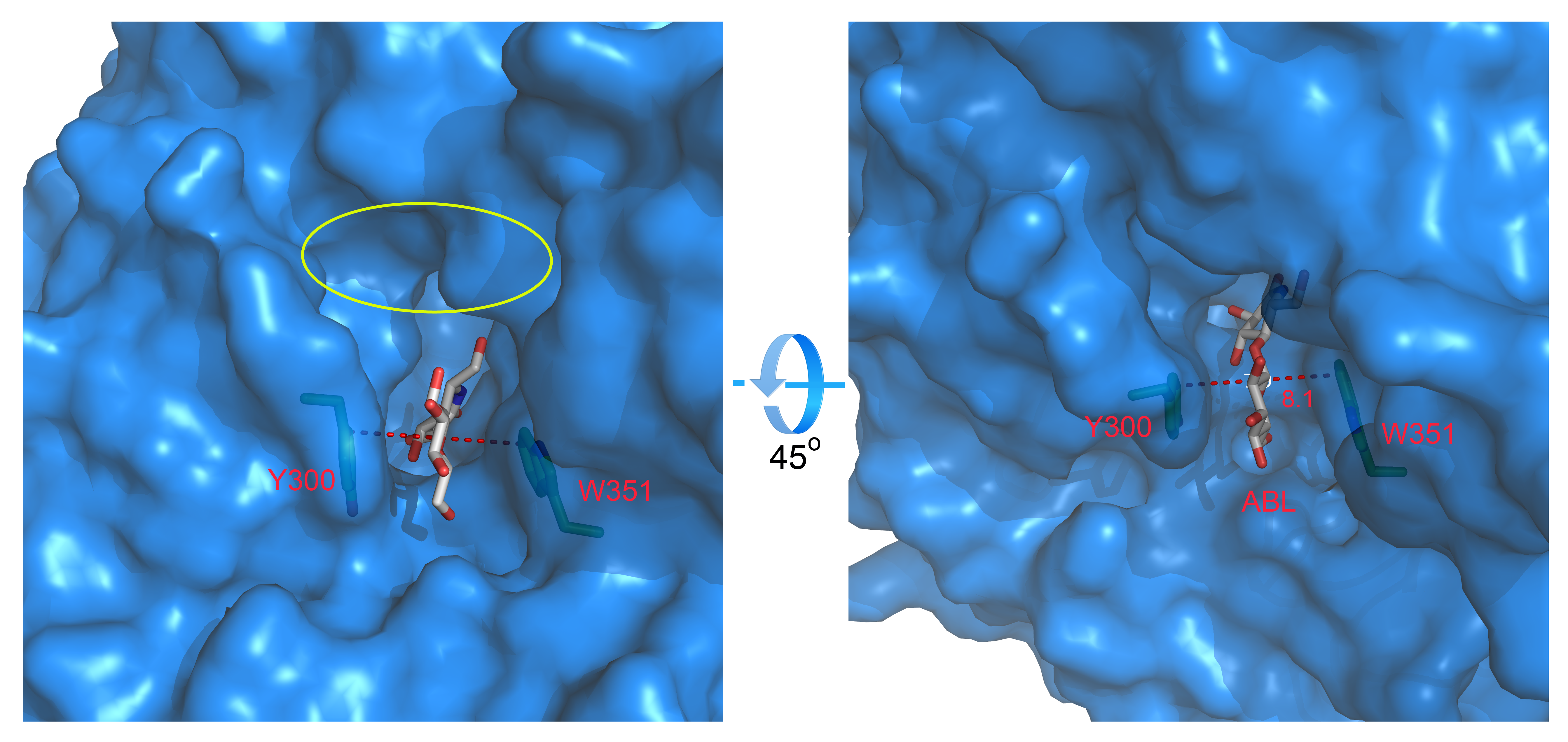

Supplement: Additional file 4: Figure S4 — A potential non-productive binding subsite. (Left): A potential binding pocket (yellow eclipse) above Y300 and W351, as seen in the GOOX-T1 structure with the presence of ABL (PDB ID: 2AXR). (Right): A 45°-rotated view, the distance between two stacking residues is 8.1 Å. [file 1754-6834-6-148-S4.tiff]
