# Supplementary material for: Xylo- and cello-oligosaccharide oxidation by gluco-oligosaccharide oxidase from Sarocladium strictum and variants with reduced substrate inhibition
Source: Biotechnol Biofuels. 2013 Oct 12;6:148. doi: 10.1186/1754-6834-6-148 (PMC4015748; doi:10.1186/1754-6834-6-148)
Supplement: Additional file 5: Table S1 — The sequences of forward oligonucleotide primers used for site-directed mutagenesis. [file 1754-6834-6-148-S5.doc]

**Table S1.** **The sequences of forward oligonucleotide primers used for site-directed mutagenesis**

| Mutation | Sequence of forward primers |
| --- | --- |
| Y72A | GGGTGGTGGTCACAGTTTTGGTTCTTATGGG |
| Y72F | GGGTGGTGGTCACAGTTTTGGTTCTTATGGG |
| E247A | CATGCGTCTTGCGATCAACGCCAATGC |
| W351A | GCGGCTGGGCTATCCAATGGGACTTCCAC |
| Q353A | CGGCTGGTGGATCGCATGGGACTTCC |
| Q353N | CGGCTGGTGGATCAATTGGGACTTCC |
| Q384A | GCTCTGGCTCTGGGCTTTCTACGACAACATCTACG |
| Q384N | GCTCTGGCTCTGGAATTTCTACGACAACATCTACG |
| N388S | GGCAGTTCTACGACAGCATCTACGACTACG |
| V38A^*^ | GACTACGACCCGGCGGCCATTGCCATC |

^*^ This primer was used on the plasmid contaning the N388S mutation to create the recombinant wild type version of GOOX-VN.
